# Supplementary material for: Shipping noise tolerance in invertebrates: A case study of the shore crab Hemigrapsus oregonensis
Source: PLoS One. 2025 Aug 12;20(8):e0329098. doi: 10.1371/journal.pone.0329098 (PMC12342237; doi:10.1371/journal.pone.0329098)
Supplement: S1 File — S1 Table. Relative Sound Pressure Level (SPL) Range at Selected Sites. S2 Table. Temperature and Salinity Measurements at Selected Sites. S3 Table. Proportion of Individual H. oregonensis That Retreated to Shelter After a Simulated Predator Attack. S4 Table. Effective sizes and variance for 71 data points from 17 studies that examined sound influences on marine arthropods’ behavior. Estimates for the current study are also included here. See the supplemental reference list below for additional information on the studies. Table S5. Mean and Standard Error of Time to Retreat to Shelter After a Simulated Predator Attack. Fig S1. Sheltering Response Time of H. oregonensis to Simulated Predator Attacks. Time taken (s) for H.oregonensis to retreat to shelter after a simulated predator attack for each treatment according to site. n = 8 per treatment per site. Fig S2. A) A funnel plot examining the heterogeneity of the data to identify potential publication biases. B) Studentized quantile-quantile (Q-Q) plot of model residuals used to examine model fit and normality of the data. (DOCX) [file pone.0329098.s001.docx]

**Supporting Information for**

**Title:**  Shipping Noise Tolerance in the Shore Crab *Hemigrapsus oregonensis*

**Authors:** Abigail Birch^1^*, Kieran D. Cox^2,3^, Kelsie A. Murchy^3^, Sandra Emry^1^, Christopher D. G. Harley^1,4^*

**Affiliations:**

^1^ Department of Zoology, University of British Columbia, Vancouver, British Columbia, Canada

^2^ Department of Biological Sciences, Simon Fraser University, Burnaby, British Columbia, Canada

^3^ Department of Biology, University of Victoria, Victoria, British Columbia, Canada

^4^ Institute for the Oceans and Fisheries, University of British Columbia, Vancouver, British Columbia, Canada

*Correspondence to: Chris Harley [harley@zoology.ubc.ca](mailto:harley@zoology.ubc.ca)

**This document Includes**

Supplemental Tables S1-S5

Supplemental Figures S1-S2

Supplemental References

**Table S1. Relative Sound Pressure Level (SPL) Range at Selected Sites**.

| Site (Lat Lon) | Relative SPL_RMS_ Range^a^ (dB re 1 μPa) |
| --- | --- |
| Acadia Beach (49.2794 N, 123.2471 W) | 109.5-115.2 |
| Brunswick Beach (49.4706 N, 123.2430 W) | 113.1-118.5 |
| Crescent Beach (49.0501 N, 122.8849 W) | 105.6-108.2 |
| Barnet Marine Park (49.2904 N, 122.9263 W) | 125.7-131.9 |
| GIW Beach (49.3026 N, 123.1262 W) | 127.9-134.2 |
| Sunset Beach (49.2781 N, 123.1383 W) | 123.4-124.7 |

^a^SPL calculated in 15-minute intervals by subtracting Root Mean Square (RMS) dB values from hydrophone sensitivity (dB re 1V/uPa).

**Table S2. Temperature and Salinity Measurements at Selected Sites**.

| Site | Temperature (°C) | Salinity (ppt) |
| --- | --- | --- |
| Acadia Beach | 8 | 24 |
| Brunswick Beach | 8 | 24 |
| Crescent Beach | 7 | 24 |
| Barnet Marine Park | 7 | 25 |
| GIW Beach | 7 | 25 |
| Sunset Beach | 9 | 25 |

**Table S3. Proportion of Individual *H. oregonensis* That Retreated to Shelter After a Simulated Predator Attack**.

| Treatment | Site | Proportion |
| --- | --- | --- |
| Control | Acadia Beach | 0.5 |
| Control | Brunswick Beach | 0.625 |
| Control | Crescent Beach | 0.375 |
| Control | Barnet Marine Park | 0.25 |
| Control | GIW Beach | 0.125 |
| Control | Sunset Beach | 0.625 |
| Noise | Acadia Beach | 0.375 |
| Noise | Brunswick Beach | 0.25 |
| Noise | Crescent Beach | 0.375 |
| Noise | Barnet Marine Park | 0.125 |
| Noise | GIW Beach | 0.25 |
| Noise | Sunset Beach | 0.625 |

**Table S4.** Effective sizes and variance for 71 data points from 17 studies that examined sound influences on marine arthropods' behavior. Estimates for the current study are also included here. See the supplemental reference list below for additional information on the studies.

| Authors | Response Type | Sound | Effect Size | Variance |
| --- | --- | --- | --- | --- |
| Wale et al. 2013 | Resource Detection | Anthropogenic | 0.1699 | 0.1549 |
|  | Anti-Predator Response | Anthropogenic | 0.8414 | 0.1470 |
|  | Righting Time | Anthropogenic | -1.2359 | 0.1856 |
| Randall Hughes et al. 2014 | Food Consumption | Bioacoustics | -0.3115 | 0.1723 |
|  | Food Consumption | Bioacoustics | -0.2696 | 0.1709 |
|  | Food Consumption | Bioacoustics | 0.1833 | 0.1686 |
|  | Food Consumption | Bioacoustics | 0.2084 | 0.1692 |
| Filiciotto et al. 2018 | Social Interactions | Tones | -0.1751 | 0.2246 |
|  | Social Interactions | Tones | -1.6988 | 0.3337 |
| Lagardere 1982 | Food Consumption | Anthropogenic | -0.0809 | 0.0401 |
|  | Food Consumption | Anthropogenic | -0.1418 | 0.0403 |
|  | Food Consumption | Anthropogenic | -0.1673 | 0.0404 |
|  | Food Consumption | Anthropogenic | -0.1987 | 0.0405 |
|  | Food Consumption | Anthropogenic | 0.1403 | 0.0403 |
|  | Food Consumption | Anthropogenic | 0.0331 | 0.0400 |
|  | Food Consumption | Anthropogenic | 0.0257 | 0.0413 |
|  | Food Consumption | Anthropogenic | -0.2128 | 0.0557 |
|  | Food Consumption | Anthropogenic | -0.3423 | 0.0890 |
|  | Food Consumption | Anthropogenic | -0.2500 | 0.0989 |
|  | Food Consumption | Anthropogenic | -0.4331 | 0.1471 |
|  | Food Consumption | Anthropogenic | -0.2945 | 0.1888 |
| Andriguetto-Filho et al. 2005 | Movement and Occurance | Anthropogenic | -0.0895 | 0.0218 |
|  | Movement and Occurance | Anthropogenic | -0.0287 | 0.0217 |
| Roberts and Laidre 2019 | Shelter and Camoflauge | Anthropogenic | -0.6749 | 0.0772 |
| Stanley et al. 2014 | Reproduction and Settlement | Anthropogenic | 3.1158 | 2.9140 |
|  | Reproduction and Settlement | Anthropogenic | 0.1937 | 0.6754 |
|  | Reproduction and Settlement | Anthropogenic | 3.8294 | 4.0612 |
|  | Reproduction and Settlement | Anthropogenic | 2.3910 | 1.9900 |
|  | Reproduction and Settlement | Anthropogenic | 0.2151 | 0.6774 |
|  | Reproduction and Settlement | Anthropogenic | 0.4157 | 0.7067 |
| Carter et al. 2020 | Shelter and Camoflauge | Anthropogenic | -0.4667 | 0.0695 |
|  | Shelter and Camoflauge | Anthropogenic | -0.4381 | 0.0629 |
|  | Anti-Predator Response | Anthropogenic | 0.4423 | 0.0690 |
|  | Anti-Predator Response | Anthropogenic | 0.4180 | 0.0626 |
| Tidau and Briffa 2024 | Shelter and Camoflauge | Anthropogenic | -0.5377 | 0.1259 |
|  | Social Interactions | Anthropogenic | 0.0309 | 0.0852 |
|  | Social Interactions | Anthropogenic | 0.1506 | 0.0984 |
| Aspirault et al. 2023 | Food Consumption | Anthropogenic | -0.0470 | 0.1668 |
|  | Food Consumption | Anthropogenic | -0.0804 | 0.1670 |
| Ruiz-Ruiz et al. 2019 | Reproduction and Settlement | Anthropogenic | 0.4905 | 0.1712 |
|  | Reproduction and Settlement | Anthropogenic | 0.3244 | 0.1642 |
| Tidau and Briffa 2019 | Shelter and Camoflauge | Anthropogenic | 0.1148 | 0.3109 |
|  | Shelter and Camoflauge | Anthropogenic | -0.6227 | 0.3301 |
|  | Shelter and Camoflauge | Anthropogenic | -0.4047 | 0.2562 |
|  | Shelter and Camoflauge | Anthropogenic | -0.0492 | 0.2681 |
| Hubert et al. 2018 | Movement and Occurance | White noise | 0.1659 | 0.0833 |
|  | Resource Detection | White noise | 0.0816 | 0.0827 |
|  | Movement and Occurance | White noise | -0.2264 | 0.0839 |
|  | Resource Detection | White noise | -0.1193 | 0.0829 |
| Day et al. 2022 | Righting Time | Anthropogenic | 0.2866 | 0.2571 |
|  | Righting Time | Anthropogenic | 0.2697 | 0.8535 |
|  | Righting Time | Anthropogenic | -0.6579 | 0.9536 |
|  | Righting Time | Anthropogenic | 0.5734 | 0.1173 |
|  | Righting Time | Anthropogenic | 0.3551 | 0.1129 |
|  | Righting Time | Anthropogenic | 0.5087 | 0.3372 |
|  | Righting Time | Anthropogenic | 0.0000 | 0.3095 |
|  | Righting Time | Anthropogenic | 0.9225 | 0.4004 |
|  | Righting Time | Anthropogenic | 0.1966 | 0.3137 |
| Azarm-Karnagh et al. 2023 | Movement and Occurance | White noise | 0.0188 | 0.0571 |
|  | Movement and Occurance | White noise | -0.0285 | 0.0572 |
|  | Resource Detection | White noise | 0.4433 | 0.0724 |
|  | Resource Detection | White noise | -0.6679 | 0.0660 |
| Celi et al. 2013 | Social Interactions | Tones | -0.3395 | 0.6933 |
|  | Social Interactions | Tones | 0.0942 | 0.6687 |
|  | Social Interactions | Tones | -0.2604 | 0.6824 |
|  | Social Interactions | Tones | 0.0793 | 0.6681 |
|  | Social Interactions | Tones | -0.3171 | 0.6899 |
|  | Social Interactions | Tones | 0.1037 | 0.6692 |
| Chan et al. 2010 | Shelter and Camoflauge | Anthropogenic | -0.4711 | 0.0718 |
|  | Shelter and Camoflauge | Anthropogenic | -0.4219 | 0.0708 |
|  | Shelter and Camoflauge | Anthropogenic | -0.3257 | 0.0578 |
| This study | Anti-Predator Response | Anthropogenic | 0.9747 | 0.4433 |
|  | Shelter and Camoflauge | Anthropogenic | -0.1859 | 0.3373 |

**Table S5. Mean and Standard Error of Time to Retreat to Shelter After a Simulated Predator Attack**.

| Treatment | Site | Mean Time to Retreat (s) | SE Time to Retreat (s) |
| --- | --- | --- | --- |
| Control | Acadia Beach | 38.5 | 8.96 |
| Control | Brunswick Beach | 32.4 | 8.80 |
| Control | Crescent Beach | 50.6 | 5.21 |
| Control | Barnet Marine Park | 53.0 | 5.20 |
| Control | GIW Beach | 57.0 | 3.00 |
| Control | Sunset Beach | 32.5 | 9.24 |
| Noise | Acadia Beach | 38.9 | 10.3 |
| Noise | Brunswick Beach | 49.9 | 6.82 |
| Noise | Crescent Beach | 38.2 | 10.6 |
| Noise | Barnet Marine Park | 57.1 | 2.88 |
| Noise | GIW Beach | 49.6 | 6.82 |
| Noise | Sunset Beach | 32.0 | 8.88 |


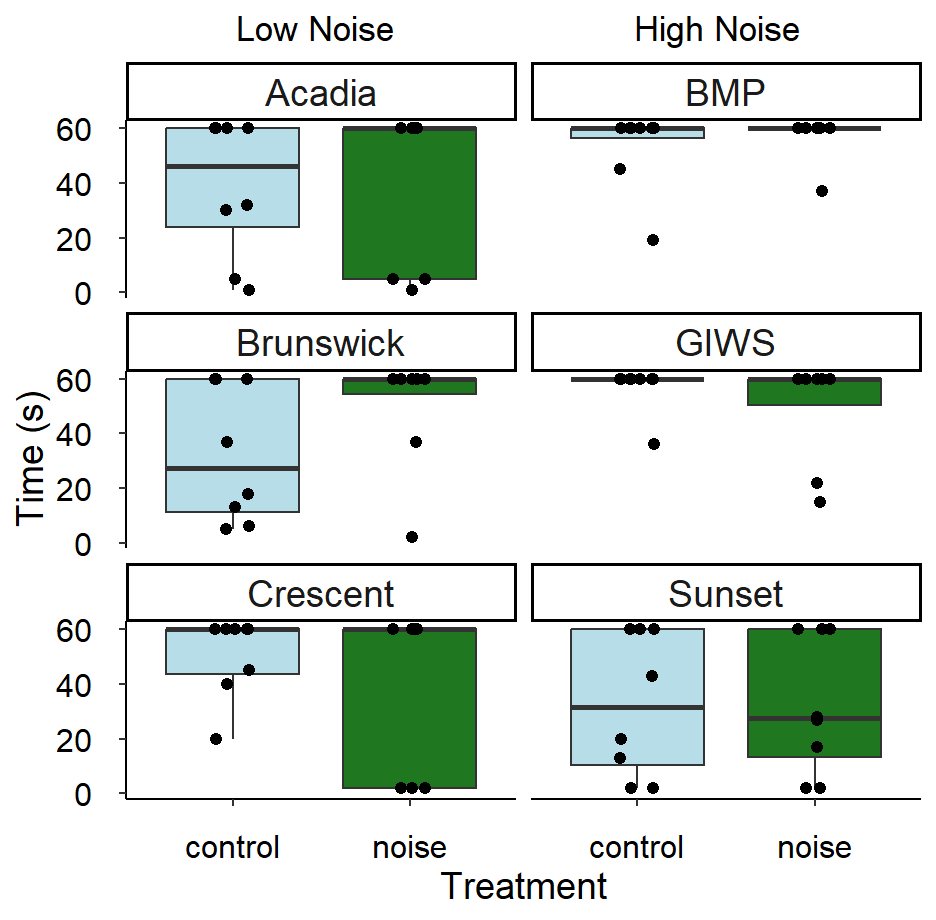


**Fig S1. Sheltering Response Time of *H. oregonensis* to Simulated Predator Attacks**. Time taken (s) for *H.oregonensis* to retreat to shelter after a simulated predator attack for each treatment according to site. N = 8 per treatment per site.


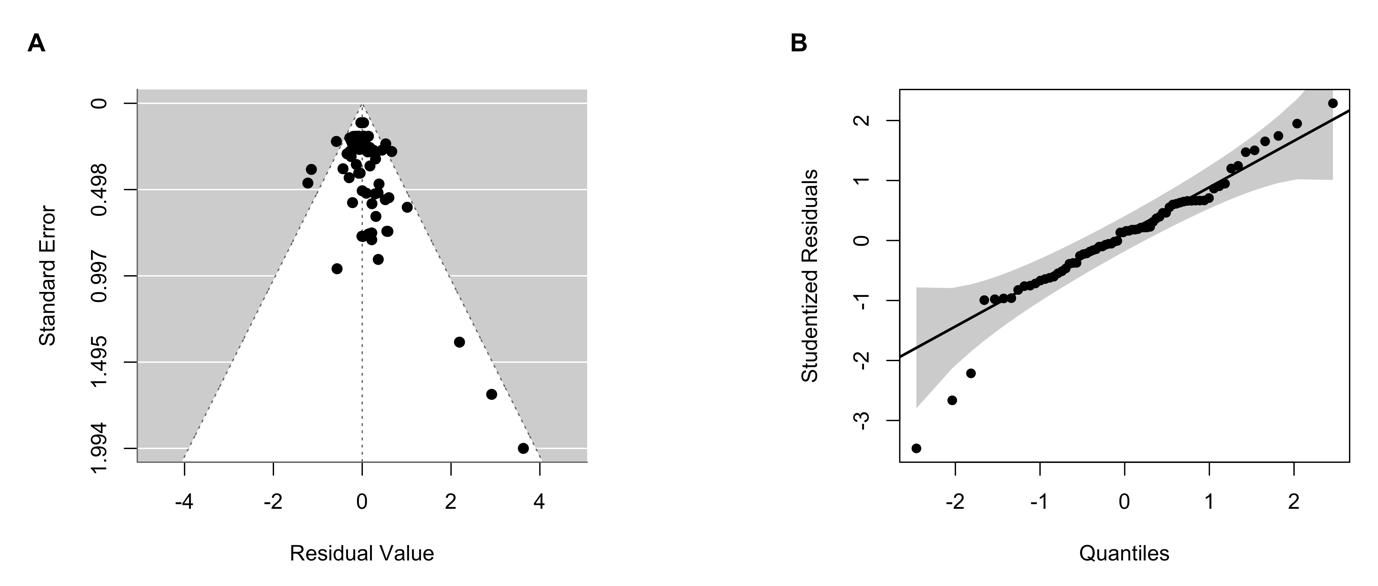
**Fig S2. A)** A funnel plot examining the heterogeneity of the data to identify potential publication biases. B) Studentized quantile-quantile (Q-Q) plot of model residuals used to examine model fit and normality of the data.

**Supplemental References**

Andriguetto-Filho JM, Ostrensky A, Pie MR, Silva UA, Boeger WA. Evaluating the impact of seismic prospecting on artisanal shrimp fisheries. Continental Shelf Research. 2005;25: 1720–1727. doi:10.1016/j.csr.2005.05.003

Aspirault A, Winkler G, Jolivet A, Audet C, Chauvaud L, Juanes F, et al. Impact of vessel noise on feeding behavior and growth of zooplanktonic species. Front Mar Sci. 2023;10: 1111466. doi:10.3389/fmars.2023.1111466

Azarm-Karnagh S, López Greco L, Shafiei Sabet S. Annoying noise: effect of anthropogenic underwater noise on the movement and feeding performance in the red cherry shrimp, Neocaridina davidi. Front Ecol Evol. 2023;11: 1091314. doi:10.3389/fevo.2023.1091314

Carter EE, Tregenza T, Stevens M. Ship noise inhibits colour change, camouflage, and anti-predator behaviour in shore crabs. Current Biology. 2020;30: R211–R212. doi:10.1016/j.cub.2020.01.014

Celi M, Filiciotto F, Parrinello D, Buscaino G, Damiano A, Cuttitta A, et al. Physiological and agonistic behavioural response of *Procambarus clarkii* to an acoustic stimulus. Journal of Experimental Biology. 2013; jeb.078865. doi:10.1242/jeb.078865

Chan AAY-H, Giraldo-Perez P, Smith S, Blumstein DT. Anthropogenic noise affects risk assessment and attention: the distracted prey hypothesis. Biol Lett. 2010;6: 458–461. doi:10.1098/rsbl.2009.1081

Day RD, Fitzgibbon QP, McCauley RD, Baker KB, Semmens JM. The impact of seismic survey exposure on the righting reflex and moult cycle of Southern Rock Lobster (Jasus edwardsii) puerulus larvae and juveniles. Environmental Pollution. 2022;309: 119699. doi:10.1016/j.envpol.2022.119699

Filiciotto F, Sal Moyano MP, De Vincenzi G, Hidalgo F, Sciacca V, Bazterrica MC, et al. Are semi-terrestrial crabs threatened by human noise? Assessment of behavioural and biochemical responses of Neohelice granulata (Brachyura, Varunidae) in tank. Marine Pollution Bulletin. 2018;137: 24–34. doi:10.1016/j.marpolbul.2018.07.023

Hubert J, Campbell J, Van Der Beek JG, Den Haan MF, Verhave R, Verkade LS, et al. Effects of broadband sound exposure on the interaction between foraging crab and shrimp – A field study. Environmental Pollution. 2018;243: 1923–1929. doi:10.1016/j.envpol.2018.09.076

Hughes AR, Mann DA, Kimbro DL. Predatory fish sounds can alter crab foraging behaviour and influence bivalve abundance. Proc R Soc B. 2014;281: 20140715. doi:10.1098/rspb.2014.0715

Lagardère  JP. Effects of noise on growth and reproduction of Crangon crangon in rearing tanks. Mar Biol. 1982;71: 177–185. doi:10.1007/BF00394627

Roberts L, Laidre ME. Finding a home in the noise: cross-modal impact of anthropogenic vibration on animal search behaviour. Biology Open. 2019;8: bio041988. doi:10.1242/bio.041988

Ruiz-Ruiz PA, Hinojosa IA, Urzua A, Urbina MA. Anthropogenic noise disrupts mating behavior and metabolic rate in a marine invertebrate. Den Haag, The Netherlands; 2019. p. 040006. doi:10.1121/2.0001302

Stanley JA, Wilkens SL, Jeffs AG. Fouling in your own nest: vessel noise increases biofouling. Biofouling. 2014;30: 837–844. doi:10.1080/08927014.2014.938062

Tidau S, Briffa M. Anthropogenic noise limits resource distribution without changing social hierarchies. Science of The Total Environment. 2024;922: 171309. doi:10.1016/j.scitotenv.2024.171309

Tidau S, Briffa M. Distracted decision makers: ship noise and predation risk change shell choice in hermit crabs. Behavioral Ecology. 2019;30: 1157–1167. doi:10.1093/beheco/arz064

Wale MA, Simpson SD, Radford AN. Noise negatively affects foraging and antipredator behaviour in shore crabs. Animal Behaviour. 2013;86: 111–118. doi:10.1016/j.anbehav.2013.05.001
