# Supplementary material for: Shipping noise tolerance in invertebrates: A case study of the shore crab Hemigrapsus oregonensis
Source: PLoS One. 2025 Aug 12;20(8):e0329098. doi: 10.1371/journal.pone.0329098 (PMC12342237; doi:10.1371/journal.pone.0329098)
Supplement: Attachment — Submitted filename: Minor revision request for pone D_25_08170.docx [file pone.0329098.s002.docx]

**General comments**

The manuscript presents results of original research, and not been published elsewhere, and written in standard English.

Experiments, statistics, and other analyses are performed to a good technical standard and are described in sufficient detail.

The research meets all applicable standards for the ethics of experimentation and research integrity.

Conclusions are presented in an appropriate fashion, supported by the data. Despite focused on a single species, it provide argumentation for reflections and further developments.

**Requests for minor revision**

Title: the title would be more catching if includes a more general aspect: “Shipping noise tolerance in invertebrates: the case of shore crab Hemigrapsus oregonensis”

Row 141: what Zoom amplifier has been used? Probably the F6? At what sampling frequency? Please clarify.

Row 158: when the 1h recordings were obtained? There should be a difference between day and night, especially when linked to living organisms’ circadian rhythms. Please clarify and add a comment on this aspect.

Fig.1 and caption: the figure shows 6 sites, even you implemented the experiments in 9 sites. You missed additional 3 high noise sites in the map (probably because far from the others?). In case, include in the caption the 3 high noise sites that are not displayed.

Fig.3 and caption: Specify that the initial response is to the simulated predator attack. Add in the caption that the feeding experiments gave 100% uniform response (this is the reason why you do not show in the figure). Again you show 6 sites instead of 9? What am I missing?

Main Issue 1: you refer to SPL, but you do not report any information about calibration of the hydrophone+zoom. Please include how calibration has been implemented and the accuracy of sensitivity across frequencies.

Main issue 2: please clarify why you choose the bird as a predator and provide a spectrum of the signal you introduced to simulate the predator attack. Samples of the spectra of the shipping noise in the 9 sites would also be welcome in the supporting information.
