# Supplementary material for: Shipping noise tolerance in invertebrates: A case study of the shore crab Hemigrapsus oregonensis
Source: PLoS One. 2025 Aug 12;20(8):e0329098. doi: 10.1371/journal.pone.0329098 (PMC12342237; doi:10.1371/journal.pone.0329098)
Supplement: Attachment — Submitted filename: Response to Reviewers PONE_D_25_08170 resubmitted.docx [file pone.0329098.s003.docx]

Journal: PLOS ONE

Manuscript ID: PONE-D-25-08170

Title: “Shipping noise tolerance in invertebrates: A case study of the shore crab *Hemigrapsus oregonensis*”

Dear Dr. Hewitt

We have revised our manuscript, PONE-D-25-08170, entitled “Shipping noise tolerance in invertebrates: A case study of the shore crab *Hemigrapsus oregonensis*.” We have addressed all the referees’ suggestions. A detailed outline of these revisions is below. We thank the reviewer for thoroughly examining our manuscript, providing constructive guidance, and recommending it for publication in *PLOS ONE.*

We found the referees’ suggestions insightful, especially those regarding necessary clarifications within our Methods section. Addressing these comments allowed us to clarify our methodological approach, enhancing both reader comprehension and the reproducibility of our study. We appreciate the reviewer’s overall positive assessment of our manuscript, and we are glad that the manuscript’s broader implications were clear despite the species-specific focus.

Having revised our manuscript per the referees’ suggestions, we hope our manuscript is now suitable for publication in *PLOS ONE*.

Thank you for your continued consideration of this manuscript.

Dr. Chris Harley

**Academic Editor Comments**

Journal requirements:

1. Please ensure that your manuscript meets PLOS ONE's style requirements, including those for file naming. The PLOS ONE style templates can be found at

<https://journals.plos.org/plosone/s/file?id=wjVg/PLOSOne_formatting_sample_main_body.pdf> and

[https://journals.plos.org/plosone/s/file?id=ba62/PLOSOne_formatting_sample_title_authors_affiliations.pdf](https://track.editorialmanager.com/CL0/https:%2F%2Fjournals.plos.org%2Fplosone%2Fs%2Ffile%3Fid=ba62%2FPLOSOne_formatting_sample_title_authors_affiliations.pdf/1/010f01967d0861b6-0543bab4-1681-453c-a725-db969eff3c7c-000000/jyWtbOYaXcGM0IFggygecCIDzWmlw8UWYM1_BuTpxS4=208)

**Author Response**

We have done our best to follow all of the style requirements, but please let us know if we missed anything.

2. To comply with PLOS ONE submissions requirements, in your Methods section, please provide additional information regarding the experiments involving animals and ensure you have included details on (1) methods of sacrifice, (2) methods of anesthesia and/or analgesia, and (3) efforts to alleviate suffering.

**Author Response**

We have revised the Methods section to clarify how animal welfare was addressed during the study. Specifically, we have added details on how stress was minimized and included a statement noting that no animals were sacrificed or subjected to invasive procedures requiring anesthesia or analgesia. The following text has been added beginning at row 189: "Tanks were filled with seawater maintained at 12 °C and 33 psu salinity to mimic natural conditions and minimize stress. Tank openings were covered to prevent crabs from escaping, and seawater was aerated using an air stone suspended from the cover. Water changes were done once per week. Crabs were fed approximately 25 g of Thrive Hermit Crab Pellet Diet once per week. Crabs were acclimated in the holding tanks for one week before experimental trials began. No invasive procedures were performed, and no animals were sacrificed during this study. All crabs were returned to their original collection sites following the conclusion of the experiment."

3. Thank you for stating the following financial disclosure:

 [Funding was provided by an NSERC Discovery Grant (RGPIN-2022-04683) to CDGH and the Liber Ero Fellowship Program to KDC. AB was supported by an Ecosystems and Oceans Science Contribution Framework grant awarded to KDC and Francis Juanes.].

Please state what role the funders took in the study.  If the funders had no role, please state: "The funders had no role in study design, data collection and analysis, decision to publish, or preparation of the manuscript." If this statement is not correct you must amend it as needed.

Please include this amended Role of Funder statement in your cover letter; we will change the online submission form on your behalf.

**Author Response**

The funders’ role has been stated in author contributions section of the manuscript.

4. In the online submission form, you indicated that [Data will be submitted to a public repository upon manuscript acceptance. We are happy to provide the data to the reviewers if requested.].

All PLOS journals now require all data underlying the findings described in their manuscript to be freely available to other researchers, either 1. In a public repository, 2. Within the manuscript itself, or 3. Uploaded as supplementary information. This policy applies to all data except where public deposition would breach compliance with the protocol approved by your research ethics board. If your data cannot be made publicly available for ethical or legal reasons (e.g., public availability would compromise patient privacy), please explain your reasons on resubmission and your exemption request will be escalated for approval.

**Author Response**

The data has been shared to the public repository Figshare. It is available through the following doi: <http://doi.org/10.6084/m9.figshare.29090081>.

6. PLOS requires an ORCID iD for the corresponding author in Editorial Manager on papers submitted after December 6th, 2016. Please ensure that you have an ORCID iD and that it is validated in Editorial Manager. To do this, go to ‘Update my Information’ (in the upper left-hand corner of the main menu), and click on the Fetch/Validate link next to the ORCID field. This will take you to the ORCID site and allow you to create a new iD or authenticate a pre-existing iD in Editorial Manager.

**Author Response**

The ORCID iD for the corresponding author is 0000-0003-4099-943X. We will be sure to update this during the resubmission process.

7. Your ethics statement should only appear in the Methods section of your manuscript. If your ethics statement is written in any section besides the Methods, please move it to the Methods section and delete it from any other section. Please ensure that your ethics statement is included in your manuscript, as the ethics statement entered into the online submission form will not be published alongside your manuscript.

**Author Response**

The ethics statement has been included in the Methods section at row 183, as follows:

*“Hemigrapsus oregonensis* were collected from the six sites between the 5^th^ and 7^th^ of January 2024 (scientific collecting license XMCFR 36 2023).” No other ethics approvals were applicable to this study.

8. We note that Figure 1B in your submission contain [map/satellite] images which may be copyrighted. All PLOS content is published under the Creative Commons Attribution License (CC BY 4.0), which means that the manuscript, images, and Supporting Information files will be freely available online, and any third party is permitted to access, download, copy, distribute, and use these materials in any way, even commercially, with proper attribution. For these reasons, we cannot publish previously copyrighted maps or satellite images created using proprietary data, such as Google software (Google Maps, Street View, and Earth). For more information, see our copyright guidelines: <http://journals.plos.org/plosone/s/licenses-and-copyright>.

We require you to either (1) present written permission from the copyright holder to publish these figures specifically under the CC BY 4.0 license, or (2) remove the figures from your submission:

1. You may seek permission from the original copyright holder of Figure 1B to publish the content specifically under the CC BY 4.0 license.

We recommend that you contact the original copyright holder with the Content Permission Form (<http://journals.plos.org/plosone/s/file?id=7c09/content-permission-form.pdf>) and the following text:

“I request permission for the open-access journal PLOS ONE to publish XXX under the Creative Commons Attribution License (CCAL) CC BY 4.0 (<http://creativecommons.org/licenses/by/4.0/>). Please be aware that this license allows unrestricted use and distribution, even commercially, by third parties. Please reply and provide explicit written permission to publish XXX under a CC BY license and complete the attached form.”

Please upload the completed Content Permission Form or other proof of granted permissions as an "Other" file with your submission.

In the figure caption of the copyrighted figure, please include the following text: “Reprinted from [ref] under a CC BY license, with permission from [name of publisher], original copyright [original copyright year].”

2. If you are unable to obtain permission from the original copyright holder to publish these figures under the CC BY 4.0 license or if the copyright holder’s requirements are incompatible with the CC BY 4.0 license, please either i) remove the figure or ii) supply a replacement figure that complies with the CC BY 4.0 license. Please check copyright information on all replacement figures and update the figure caption with source information. If applicable, please specify in the figure caption text when a figure is similar but not identical to the original image and is therefore for illustrative purposes only.

**Author Response**

Figure 1B and the map images were made using publicly available data from the following source <https://bcmca.ca/>.

9. Please review your reference list to ensure that it is complete and correct. If you have cited papers that have been retracted, please include the rationale for doing so in the manuscript text, or remove these references and replace them with relevant current references. Any changes to the reference list should be mentioned in the rebuttal letter that accompanies your revised manuscript. If you need to cite a retracted article, indicate the article’s retracted status in the References list and also include a citation and full reference for the retraction notice.

**Author Response**

The reference list has been reviewed and is complete and correct.

**Reviewer 1 Comments to the Author:**
**General Comment:**

The manuscript presents results of original research, and not been published elsewhere, and written in standard English.

Experiments, statistics, and other analyses are performed to a good technical standard and are described in sufficient detail.

The research meets all applicable standards for the ethics of experimentation and research integrity.

Conclusions are presented in an appropriate fashion, supported by the data. Despite focused on a single species, it provide argumentation for reflections and further developments.

**Author Response**

We thank Reviewer 1 for their positive assessment of our manuscript and for recognizing the quality of our experimental design, statistical analysis, and conclusions. We appreciate the encouraging comments regarding the integrity of the research, and we are glad that the manuscript’s broader implications were clear despite the species-specific focus.

**Requests for minor revision**

**Reviewer Comment**

Title: the title would be more catching if includes a more general aspect: “Shipping noise tolerance in invertebrates: the case of shore crab Hemigrapsus oregonensis”

**Author Response**

We appreciate the reviewer’s suggestion to broaden the title’s appeal. In response, we have revised the title to highlight the relevance of our findings to invertebrates more generally. The new title reads: “Shipping noise tolerance in invertebrates: A case study of the shore crab *Hemigrapsus oregonensis*”

**Reviewer Comment**

Row 141: what Zoom amplifier has been used? Probably the F6? At what sampling frequency? Please clarify.

**Author Response**

We used a zoom recorder, H1 handy recorder to save the wav files to an SD card. We added

clarifying details. Line 143-144: “…connected to a ZOOM recorder (H1 Handy Recorder, sampling rate = 48 kHz, 16-bit)…”

**Reviewer Comment**

Row 158: when the 1h recordings were obtained? There should be a difference between day and night, especially when linked to living organisms’ circadian rhythms. Please clarify and add a comment on this aspect.

**Author Response**

We thank the reviewer for pointing this out. We have clarified the timing of the recordings in the Methods section on Row 140 which now reads: “At each site, a one-hour recording was made between 09:30 and 17:00 using an underwater hydrophone (Cetacean Research Technology SQ-26; sensitivity: 169 dB re 1 V/µPa; frequency range: 0.020-50 kHz) connected to a ZOOM recorder (H1 Handy Recorder) at one meter depth.” We also revised Row 158 to: “**Fig 1.** **Acoustic Analysis of Study Sites in the Lower Mainland, BC**. (A) Relative sound pressure levels at selected sites based on one-hour underwater recordings made between 09:30 and 17:00 using a SQ-26 hydrophone and ZOOM recorder.”

**Reviewer Comment**

Fig.1 and caption: the figure shows 6 sites, even you implemented the experiments in 9 sites. You missed additional 3 high noise sites in the map (probably because far from the others?). In case, include in the caption the 3 high noise sites that are not displayed.

**Author Response**

We thank the reviewer for this comment. To clarify, although nine sites were initially surveyed to assess ambient noise levels, only six were ultimately included in the experimental component of the study, based on their relative SPL values and the presence of suitable crab populations. We have revised the first sentence of the Methods section to make the site selection process clearer. As the final study was implemented at only six sites, we have chosen to display only these in Figure 1.

**Reviewer Comment**

Fig.3 and caption: Specify that the initial response is to the simulated predator attack. Add in the caption that the feeding experiments gave 100% uniform response (this is the reason why you do not show in the figure). Again you show 6 sites instead of 9? What am I missing?

**Author Response**

We have now included a brief mention of the uniform feeding disruption response in the caption, which was why the data are not shown in the figure. The initial response to the simulated predator attack is already specified in the figure caption. Regarding the number of sites, the experiment was only implemented at six sites, not nine. We have clarified this in the Methods section to avoid any confusion.

**Reviewer Comment**

Main Issue 1: you refer to SPL, but you do not report any information about calibration of the hydrophone+zoom. Please include how calibration has been implemented and the accuracy of sensitivity across frequencies.

**Author Response**

We used the hydrophone sensitivity provided by the manufacturer, but did not calibrate prior to

deployment. This was consistent to what we found in the literature for these hydrophones, see Lillis

et al. 2018 *Drifting hydrophones as an ecologically meaningful approach to underwater soundscape measurement in coastal benthic habitats*, so we feel that this is justified. We changed the language to relative SPL, to account for the variation that could be present. Note that we have recently purchased a pistonphone that could be used to calibrate post-hoc if required. However, we added more details on the hydrophone, including the frequency accuracy across frequencies. Lines 141-143: “…underwater hydrophone (Cetacean Research Technology SQ-26; sensitivity: -

169 dB re 1 V/µPa; flat frequency response (± 1 dB) up to 28 kHz, frequency range: 0.020-50

kHz)”

**Reviewer Comment**

Main issue 2: please clarify why you choose the bird as a predator and provide a spectrum of the signal you introduced to simulate the predator attack. Samples of the spectra of the shipping noise in the 9 sites would also be welcome in the supporting information.

**Author Response**

Thank you for the comment. We have added a point to Row 223 to clarify that the shore bird was chosen because it is a common predator of *H. oregonensis*. To clarify, the predator attack itself did not involve noise; rather, shipping noise was played in the background to test its effect on the crab’s behavior, specifically its time to retreat to shelter. The spectrum of the shipping noise used in the experiment can be seen in Figure 2.

Regarding the sound data, we only included the SPL data for the six sites selected for the study, as the other three sites either had incomplete SPL data or did not have crabs, making them unsuitable for inclusion. This information is provided in Table S1 of the supplementary materials.
